# Endogenous endophthalmitis due to *Roseomonas mucosa* presenting as a subretinal abscess

**DOI:** 10.1186/s12348-017-0123-6

**Published:** 2017-01-28

**Authors:** Muna Bhende, Aashraya Karpe, Sukanya Arunachalam, K. Lily Therese, Jyotirmay Biswas

**Affiliations:** Sankara Nethralaya, Chennai, India

**Keywords:** *Roseomonas mucosa*, Subretinal abscess, Endogenous endophthalmitis, Vitrectomy

## Abstract

**Background:**

Endogenous bacterial endophthalmitis is an infrequently reported entity. Although *Roseomonas mucosa* has been reported to cause systemic infections in immunosuppressed individuals, ocular infection due to *Roseomonas* has been rarely reported in literature previously.

**Findings:**

A 74-year-old diabetic was diagnosed to have *Klebsiella* urinary tract infection and septicemia following which he developed ocular pain and redness. Further investigation revealed endophthalmitis with subretinal abscess and retinal detachment. The patient underwent pars plana vitrectomy with drainage of the abscess and silicone oil tamponade. The subretinal aspirate was found to contain *R. mucosa* confirmed on culture and PCR.

**Conclusion:**

Microbiological evaluation of the subretinal purulent material revealed pink-colored colonies. Nested PCR was positive for detection of the eubacterial genome as well as for detection of the *Mycobacterium tuberculosis* genome (Ref)-targeting MPB64 gene. PCR examination of the subretinal pus sample ruled out *M. tuberculosis* and confirmed *R. mucosa*. The occurrence of *Roseomonas* endogenous endophthalmitis presenting as a subretinal abscess has not yet been reported in English literature so far to the best of our knowledge.

## Introduction

Endogenous bacterial endophthalmitis is a rare entity with an incidence of 2 to 8% of all cases of endophthalmitis [1–4]. *Roseomonas mucosa* is an extremely uncommon cause of endophthalmitis with scarce reports in literature. We report a subretinal abscess in an elderly diabetic male, presumably endogenous, caused by *R. mucosa* and its successful management.

## Case report

A 74-year-old diabetic gentleman presented with redness, pain, and diminution of vision in the left eye since 2 weeks. There was no history of trauma or ocular surgery. Past history was significant for hospitalization for urinary tract infection and septicemia during which he developed the ocular symptoms. The urine and blood cultures were positive for *Klebsiella* species—further identification was not available. The patient had received one intravitreal injection of ceftazidime (2.25 mg/0.1 ml) in the left eye and was on oral cefotaxime (500 mg TDS) and topical moxifloxacin 0.5%, tobramycin 0.3%, homatropine hydrobromide 2%, and prednisolone acetate 1% eye drops.

**Fig. 1:**
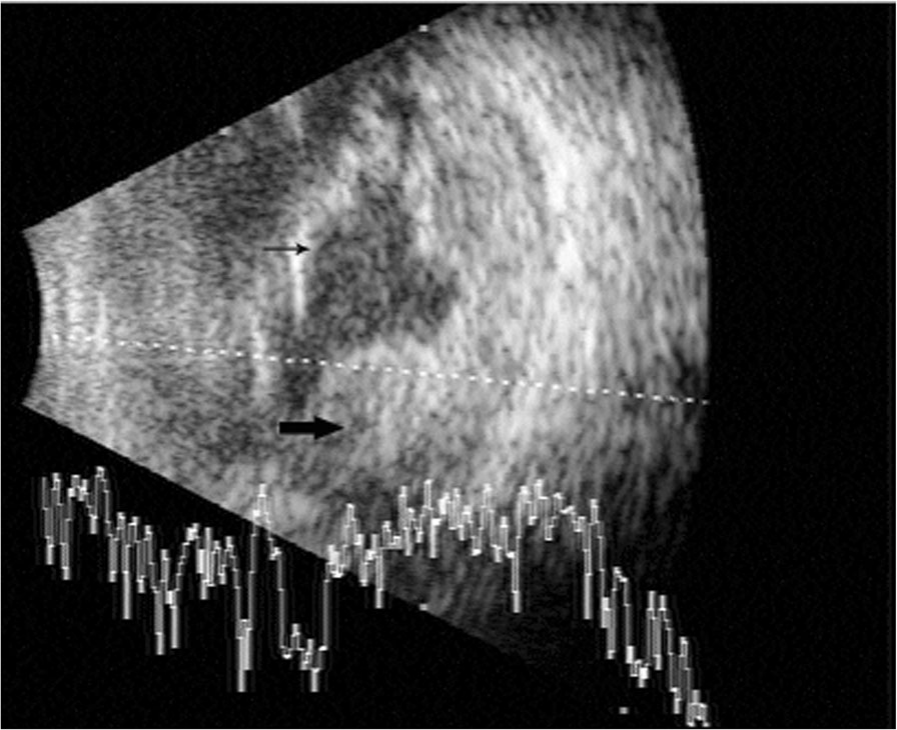
B-scan ultrasound. A vector showing vitreous exudates, the retinal detachment (*thin arrow*), and subretinal abscess (*thick arrow*)

On examination, best corrected visual acuity in the right eye was 6/6; N6 and left eye was counting fingers close to face. Examination of the right eye was unremarkable. The left eye had a mid dilated sluggishly reacting pupil, hypopyon, and nuclear sclerosis grade 3. Intraocular pressure was 15 mmHg. Vitreous exudates were present, and fundus details were unclear. B-scan echography revealed moderate number of vitreous echoes, serous choroidal detachment, and a subretinal abscess in the superotemporal quadrant (Fig. 1). The patient underwent anterior chamber tap, and intravitreal ceftazidime (2.25 mg/0.1 ml) was given. Preliminary microbiological study (Grams and KOH stains, bacterial and fungal culture) showed numerous pus cells, but no organisms. The nested PCR for detection of eubacterial genome (Ref) was positive. Since the patient’s poor systemic condition prevented immediate surgical intervention, intravitreal injection of vancomycin (1 mg/0.1 ml) and ceftazidime (2.25 mg/0.1 ml) were repeated twice on alternate days. Although mild improvement in inflammation was noted, an increase in the extent of retinal detachment was seen and the patient was posted for vitreous surgery. Intraoperatively after lensectomy and clearing of vitreous exudates, the temporal retina was seen to be detached with a large subretinal abscess with an area of overlying retinal necrosis. The abscess was drained through this area with a 20-g tapered extrusion needle attached to a 2-cc syringe, and pus was sent for microbiological study, along with a vitreous aspirate. The necrotic area was then treated with diathermy, and necrotic tissue was excised. Further drainage of the subretinal purulent material was facilitated with the use of perfluorocarbon liquid. At conclusion, a perfluorocarbon fluid-air exchange was performed, and after flattening of the retina, laser photocoagulation was performed around the retinectomy site and 360° in the periphery. 5000CS silicone oil was used to replace the air, and sclerotomies were closed. Intravitreal ceftazidime in half the dosage was injected. Postoperatively, the patient was put on oral ciprofloxacin 500 mg BD for 5 days and topical homatropine, prednisolone, moxifloxacin, and tobramycin drops. He was also under care of an internist to monitor his blood sugar levels.

**Fig. 2:**
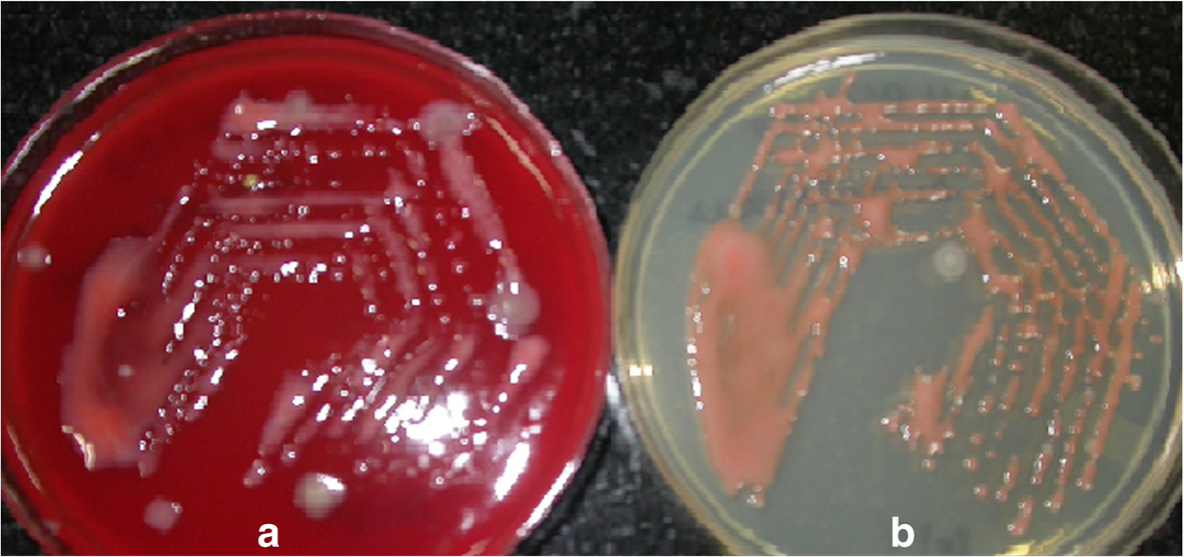
Pink-colored mucus secreting *Roseomonas mucosa* colonies on **a** blood agar and **b** Mueller-Hinton agar plates

**Fig. 3:**
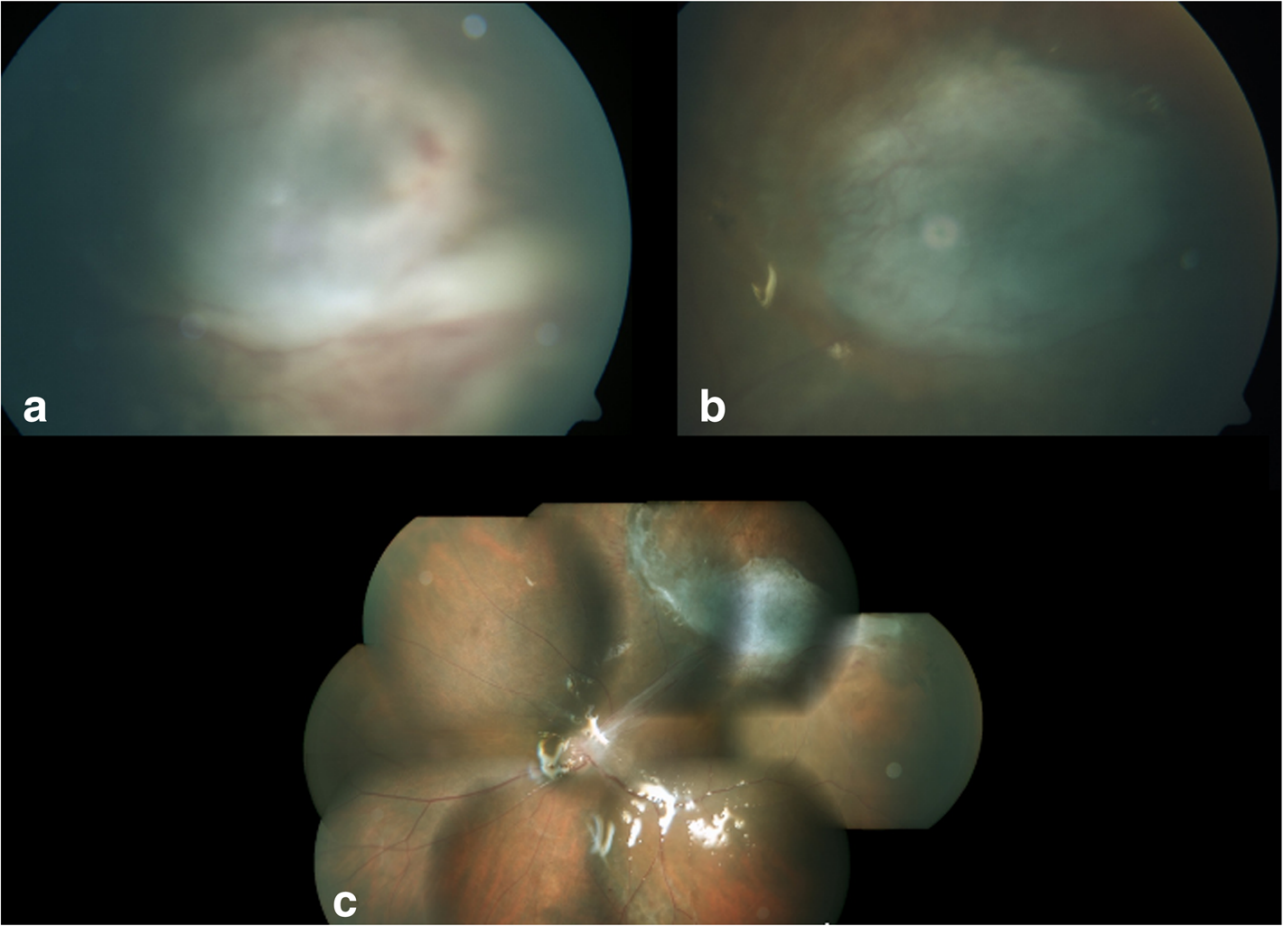
Fundus photographs. **a** Postoperative day 5, **b** postoperative day 17, **c** 8 weeks postoperative. The pictures show gradual resolution of the abscess and conversion into a flat scar. The montage picture shows a pale disc with traction band extending to the superotemporal fibrous scar

The microbiology report on the direct smears of the pus by Gram stain and Zeihl-Neelsen’s stain for acid-fast bacilli showed Gram-negative bacilli and acid-fast granules, respectively. Nested PCR was positive for detection of eubacterial genome as well as for detection of *Mycobacterium tuberculosis* genome (Ref)-targeting MPB64 gene. The vitreous sample did not show any organisms on the smear or growth on the culture; the subretinal aspirate revealed growth of *R. mucosa* with typical pink-colored mucous colonies (Fig. 2). This was confirmed by PCR-based DNA sequencing targeting the 16S ribosomal RNA gene. Culture was negative for isolation of *M. tuberculosis* by MGIT 460 liquid culture system. Postoperatively, anterior segment fibrin was noted which cleared by the end of the first week. The retina was attached with a pale disc and a fibrotic scar at the site of the abscess (Fig. 3a, b). The patient was also seen by a chest physician to rule out the possibility of a tuberculous infection, but the need for specific anti-tuberculous treatment was ruled out. Postoperatively, 3 weeks after the surgery, the patient developed angina pectoris. He was diagnosed to have an inferior wall infarct and received a stent. Systemically, he stabilized, and 8 weeks postoperatively, the vision had improved to 3/60 in the left eye, with a quiet eye and intraocular pressure of 2 mmHg. The disc was pale, and there was a shallow temporal retinal detachment caused by traction and fibrosis from the scar (Fig. 3c). No further intervention was advised.

## Discussion

Endogenous endophthalmitis is an uncommon entity [5], and subretinal abscess as a prominent feature is infrequently reported in literature. The most common organism reported to cause subretinal abscess is *Nocardia* [6–8], more so in immunocompromised patients. There are case reports of other organisms such as *Pseudomonas aeruginosa* [9], *Streptococcus viridans* [10], and *Klebsiella pneumoniae* [11] causing subretinal abscess.

The bacterial genus *Roseomonas* was first reported by Rihs et al. [12] in 1993 though the natural reservoir of the organism is unclear. *Roseomonas* genus is typically described as a slow-growing, pink, pigmented, aerobic, Gram-negative bacteria. This genus includes ten species, one of which is *R. mucosa*. This organism has been incriminated as the causative agent in catheter-related bacteremia. Literature reports have shown various instances where it has been isolated from blood, urine, and catheter samples [8, 13–17]. However, there is no report of *R. mucosa* being isolated from a subretinal abscess.

A case reported by Chen et al. [18] in 2008 showed that *Roseomonas* was the causative organism in the case of chronic postoperative endophthalmitis in an 83-year-old patient. However, in their report since the sample was not preserved, further subtyping of the species was not done. The final visual outcome was unsatisfactory.

The antibiotic sensitivity pattern of *Roseomonas* has been studied by Han et al. [19] in 2003. The choice of treatment for *Roseomonas* species is difficult as the susceptibility varies among different species with *R. mucosa* showing the highest risk of resistance. The organism has been found to be sensitive to broad-spectrum antibiotics including aminoglycosides, ciprofloxacin, imipenem, and ticarcillin but resistant to third- or fourth-generation cephalosporins such as ceftazidime [12, 14, 19].

Our case is unique in that the organism was found to be different from that predicted based on the clinical condition. PCR helped identify the organism [20, 21]. Almost complete drainage of the purulent material probably also contributed to the satisfactory outcome.

*R. mucosa* is a rare cause of endophthalmitis, and this is the first time the organism has been isolated from a subretinal abscess. Internal drainage of the abscess with identification of the organism and treatment with the appropriate antibiotics led to a satisfactory outcome in this patient.

## References

[1] Bohigian GM, Olk RJ (1986). Factors associated with a poor visual result in endophthalmitis. Am J Ophthalmol.

[2] Diamond JG (1981). Intraocular management of endophthalmitis. A systematic approach. Arch Ophthalmol.

[3] Rowsey JJ, Newsom DL, Sexton DJ, Harms WK (1982). Endophthalmitis: current approaches. Ophthalmology.

[4] Shrader SK, Band JD, Lauter CB, Murphy P (1990). The clinical spectrum of endophthalmitis: incidence, predisposing factors, and features influencing outcome. J Infect Dis.

[5] Harris EW, D’Amico DJ, Bhisitkul R, Priebe GP, Petersen R (2000). Bacterial subretinal abscess: a case report and review of the literature. Am J Ophthalmol.

[6] Ferry AP, Font RL, Weinberg RS, Boniuk M, Schaffer CL (1988). Nocardial endophthalmitis: report of two cases studied histopathologically. Br J Ophthalmol.

[7] Gregor RJ, Chong CA, Augsburger JJ, Eagle RC, Carlson KM, Jessup M (1989). Endogenous Nocardia asteroides subretinal abscess diagnosed by transvitreal fine-needle aspiration biopsy. Retina Phila Pa.

[8] Mamalis N, Daily MJ, Ross D (1988). Presumed intraocular nocardiosis in a cardiac-transplant patient. Ann Ophthalmol.

[9] Webber SK, Andrews RA, Gillie RF, Cottrell DG, Agarwal K (1995). Subretinal Pseudomonas abscess after lung transplantation. Br J Ophthalmol.

[10] Rimpel NR, Cunningham ET, Howes EL, Kim RY (1999). Viridans group Streptococcus subretinal abscess. Br J Ophthalmol.

[11] Yarng SS, Hsieh CL, Chen TL (1997). Vitrectomy for endogenous Klebsiella pneumoniae endophthalmitis with massive subretinal abscess. Ophthalmic Surg Lasers.

[12] Rihs JD, Brenner DJ, Weaver RE, Steigerwalt AG, Hollis DG, Yu VL (1993). Roseomonas, a new genus associated with bacteremia and other human infections. J Clin Microbiol.

[13] Dé I, Rolston KVI, Han XY (2004). Clinical significance of Roseomonas species isolated from catheter and blood samples: analysis of 36 cases in patients with cancer. Clin Infect Dis Off Publ Infect Dis Soc Am.

[14] Lewis L, Stock F, Williams D, Weir S, Gill VJ (1997). Infections with Roseomonas gilardii and review of characteristics used for biochemical identification and molecular typing. Am J Clin Pathol.

[15] Boyd MA, Laurens MB, Fiorella PD, Mendley SR (2012). Peritonitis and technique failure caused by Roseomonas mucosa in an adolescent infected with HIV on continuous cycling peritoneal dialysis. J Clin Microbiol.

[16] Al-Anazi KA, AlHashmi H, Abdalhamid B, AlSelwi W, AlSayegh M, Alzayed A (2013). Roseomonas bacteremia in a recipient of an allogeneic hematopoietic stem cell transplantation. Transpl Infect Dis Off J Transplant Soc.

[17] Bard JD, Deville JG, Summanen PH, Lewinski MA (2010). Roseomonas mucosa isolated from bloodstream of pediatric patient. J Clin Microbiol.

[18] Chen K-J, Lai C-C, Kuo Y-H, Wu W-C, Chen T-L (2009). Chronic postoperative Roseomonas endophthalmitis. J Clin Microbiol.

[19] Han XY, Pham AS, Tarrand JJ, Rolston KV, Helsel LO, Levett PN (2003). Bacteriologic characterization of 36 strains of Roseomonas species and proposal of Roseomonas mucosa sp nov and Roseomonas gilardii subsp rosea subsp nov. Am J Clin Pathol.

[20] Therese KL, Anand AR, Madhavan HN (1998). Polymerase chain reaction in the diagnosis of bacterial endophthalmitis. Br J Ophthalmol.

[21] Therese KL, Jayanthi U, Madhavan HN (2005). Application of nested polymerase chain reaction (nPCR) using MPB 64 gene primers to detect Mycobacterium tuberculosis DNA in clinical specimens from extrapulmonary tuberculosis patients. Indian J Med Res.

